# Scientists Popularizing Science: Characteristics and Impact of TED Talk Presenters

**DOI:** 10.1371/journal.pone.0062403

**Published:** 2013-04-30

**Authors:** Cassidy R. Sugimoto, Mike Thelwall, Vincent Larivière, Andrew Tsou, Philippe Mongeon, Benoit Macaluso

**Affiliations:** 1 School of Library and Information Science, Indiana University Bloomington, Bloomington, Indiana, United States of America; 2 School of Technology, University of Wolverhampton, Wolverhampton, United Kingdom; 3 École de bibliothéconomie et des sciences de l’information, Université de Montréal, Montréal, Canada; 4 Observatoire des Sciences et des Technologies (OST), Centre Interuniversitaire de Recherche sur la Science et la Technologie (CIRST), Université du Québec à Montréal, Montréal, Canada; Bar-Ilan University, Israel

## Abstract

The TED (Technology, Entertainment, Design) conference and associated website of recorded conference presentations (TED Talks) is a highly successful disseminator of science-related videos, claiming over a billion online views. Although hundreds of scientists have presented at TED, little information is available regarding the presenters, their academic credentials, and the impact of TED Talks on the general population. This article uses bibliometric and webometric techniques to gather data on the characteristics of TED presenters and videos and analyze the relationship between these characteristics and the subsequent impact of the videos. The results show that the presenters were predominately male and non-academics. Male-authored videos were more popular and more liked when viewed on YouTube. Videos by academic presenters were more commented on than videos by others and were more liked on YouTube, although there was little difference in how frequently they were viewed. The majority of academic presenters were senior faculty, males, from United States-based institutions, were visible online, and were cited more frequently than average for their field. However, giving a TED presentation appeared to have no impact on the number of citations subsequently received by an academic, suggesting that although TED popularizes research, it may not promote the work of scientists within the academic community.

## Introduction

Scientific communication, previously conducted through print, radio, and television media, is increasing finding outlets online [1]. While some sources merely create an online version of materials previously published in print, other venues actively aim to take advantage of the opportunities offered by web platforms [2]. For example, the *Journal of Visualized Experiments*, which recently published its 2000^th^ article, publishes lab experiments that have been professionally videographed alongside scientific descriptions, diagrams, and citations [3]. Other journals, including *The Journal of Number Theory* and *Nature*, have used YouTube to provide supplementary information for their articles [4].

In this vein, TED may be one of the most successful online producers of scientific and technical videos. TED began in 1984 as a conference dedicated to discussions of technology, entertainment, and design, but expanded to a global market in 2006 when it began hosting videos of the conference talks (TED Talks) on its own website (as well as on YouTube). On November 13, 2012, TED announced that it had reached its billionth video view [5]. Other measures demonstrate the success of TED Talks at popularizing science; although it also includes entertainment-related videos, the TED Talks website is the fourth most popular technology website in the world [6] and the most popular conference and events website [7]. These statistics reflect a wide public interest in scientific knowledge; a 2012 survey reported that more than 90% of Americans are moderately or very interested in new scientific discoveries, with the Internet representing the main source of information for learning about such discoveries [8].

Scientific communication has been linked to more informed civil discourse and greater public participation in policymaking [9]. However, despite keen public interest in science, multiple nations report that their populations are lacking in basic factual knowledge about science [8]. This begs the question of *where* and *from whom* people are gathering their information about science and technology. Confidence in scientists is high (rivalled in the U.S. only by military leaders), and the public confers immense prestige on these individuals [8]. However, there is a Janus-faced nature to the public’s perception of scientific authority, which ranges from “infallible” to “isolated, arrogant, obscure…[and] unethical” [10]. Nisbet et al. reviewed popular characterizations of scientists (e.g., evil, easily manipulated, eccentric, elite, and mysterious); in nearly all of the characterizations, scientists are perceived to be outside of the normal boundaries of society, either positively (e.g., akin to a priest) or negatively (e.g., the “mad scientist”) [11]. These perceptions are often mediated through journalists in news media or popular science magazines and programmes. The relationship between scientists and the public is thereby influenced by the perception of the media used to communicate science [12].

There is a widespread belief that “scientists have a basic responsibility to interact with the public” [13], while some within academe suggest that popularization should be a secondary activity (which, incidentally, brings into question the academic’s reputation and motivations) [13], [14], [15]. Attempts to effectively disseminate scientific information to the lay public is often complicated by a variety of factors, including the highly technical language of scientific information, the qualified presentation of scientific results, the lack of training in popularization, and the lack of adequate rewards [16]. One such reward could be an increase in “scientific capital” [17] with webometric indicators providing an indicator of capital accumulated outside the scientific world and citations as capital from within the academic sphere. Previous studies have shown that senior scholars and the scientific elite are more likely to undertake the task of popularization than their less experienced (or renowned) counterparts [14], [15], [18], [19]; nevertheless, recent research suggests a democratization of participation in popularization [15].

Disseminating scientific information to the public is difficult; Boulter asserted that “the public finds much of the detail of science unintelligible” and that scholars “need to be aware of their changing status and of the need to respond to the public’s demand for more openness” [10]. There is also a burden in communicating “what science can and cannot do” [10] and making transparent processes such as revisions and retractions [20], [21]. In addition, certain segments of the population believe that science is not objective, but rather used for political or economic purposes [22], [23]. This is particularly problematic when scientific theories become associated with a few outspoken individuals [24]. The situation is further exacerbated in the meticulously curated world of TED Talks [25], where the work of a single individual (and often the larger field of which they are a part) is condensed into 18 minutes or less of “episodic framing” [26].

Although visualizations have long been an important aspect of scientific communication, helping both to persuade and elucidate [27], [28], in recent years scientists have increasingly turned to infographics and other visualization methods in order to communicate their ideas to the lay public, spurred by the Internet and sites such as TED (and perhaps typified by the performances of Hans Rosling, called a “master of science communication” [29]). A vital component of TED Talks is the entertainment aspect (as evidenced by its placement in the conference’s name), and one means of packaging scientific talks as "entertainment" is to appropriate the methods used by professional “entertainers.” For example, the use of satire, humor, and other forms of comedy (i.e., the rhetorical devices used in many entertainment platforms) may help the public to engage with science [30]. Indeed, “the popularity of scientific claims is inevitably defined by the available technology and preferred aesthetics of contemporary media” [31], and accordingly, it should not be surprising that the visual possibilities offered by the Internet have been embraced by knowledge disseminators.

Given that TED Talks are an example of popular scientific communication, it is important to understand what types of people present at TED, as well as to determine if these individuals are particularly successful from the perspectives of popular and academic receipt. Information regarding the presenters can provide insights into the credentials of those who are disseminating scientific information on an almost unprecedented scale and the degree to which the viewing public prefers videos presented by academics to those presented by non-academics (or vice-versa). This research therefore seeks to answer the following general questions:

1. What are the characteristics of academic TED Talk presenters?
2. What is the relationship (if any) between presenter characteristics and video popularity metrics?
3. What impact does giving a TED Talk have on the citation impact of the academic presenters?

The answers to these questions can provide valuable information to those educators and policy makers charged with evaluating the public dissemination and consumption of science [12]. The findings can also be useful to those who seek to imitate TED’s success in widely communicating science to the public. A workshop of researchers on science communication concluded that “the greatest challenge to science communication online remains simply *reaching audiences*” [32]. With more than one billion views to its credit, the TED Talks website seems to have overcome this obstacle and represents a highly successful form of science popularization. Nevertheless, little is known regarding the demographic and academic qualities of presenters, the reactions of the audience in regards to the presenters’ characteristics, and the relationship between these characteristics and the popular appeal of associated videos. Furthermore, no research has been conducted to evaluate the extent to which engaging in science communication results in increased academic capital (in terms of citation impact) for the presenters. This work provides an initial exploration into these issues.

## Methods

This paper builds upon a previous analysis of impact metrics for TED Talks [16]. The present study uses the same list of 1,202 videos (representing a comprehensive list of TED Talks available on YouTube and the TED website in April 2012) but updates the popularity metrics (e.g., number of views, comments, and proportion of ‘likes’ to ‘dislikes’ on YouTube) with new data culled in late 2012. Specifically, YouTube data were gathered via the YouTube API for video statistics on November 28, 2012, while data pertaining to videos hosted by TED were gathered by automatically extracting information from the video home pages on TED’s website (this latter process was conducted between November 20, 2012 and December 2, 2012).

Data for each of the presenters were then gathered and combined with information regarding their respective videos. A list of unique individual presenters was created by removing duplicate presenters (i.e., those who presented more than one TED Talk), groups of presenters (e.g., Improv Everywhere, They Might Be Giants), fictional presenters (e.g., Jor-El), and animals (e.g., Einstein the Parrot). The remaining 998 presenters were coded for perceived gender and academic status. Perceived gender was coded by examining both the videos and the pronouns used in the biographical material provided on the TED website. Academic status was coded as either “academic” or “non-academic.” An academic was operationalized as a presenter who had earned a doctoral degree and was affiliated with an academic institution. Individuals whose doctoral degrees were in progress were not coded as academics. The distinction between academics and researchers in other sectors was informed by previous studies which showed high levels of public trust in university researchers relative to their private and governmental counterparts [33], [34].

Further demographic information for “academics” was then gathered (i.e., date of doctoral degree, current academic affiliation, and academic rank) through biographical sources, university websites, and online curriculum vitae. Rankings from the Times Higher Education (THE) World University Rankings 2011–2012 were used to rank the universities with which the academics were affiliated [35]. The online presence for each academic was also measured; that is, the degree to which the scholar was visible to the public via maintaining a website, making a CV available, and engaging in social media activities. This was accomplished via web searches, searching the English version of Wikipedia, CV analysis, and examination of scholars’ websites. Mainstream media presence was evaluated by examining CVs, websites, and general web searches. The searches were limited to the first two pages of Google results, although multiple searches were conducted if relevant information was not available elsewhere; for example, if a simple search for “John Doe” turned up a personal webpage but *not* a Twitter account (and if an examination of the personal webpage did not produce a Twitter link), then a second search was conducted using the search string “John Doe twitter.” In addition, if the presenter had a relatively common name (or other difficulties that arose during the search process), information provided on the TED site was used in order to qualify the searches. For example, if an individual’s university affiliation was provided by TED, this data would be added to the search string if necessary.

The publication record of each TED presenter was compiled using Thomson Reuters’ Web of Science for 1980–2011. All papers on which the name of TED presenters appeared were retrieved, without any restriction on the country or document type; the papers returned by this process included many homonyms. TED presenters’ publication records were then cleaned by removing papers authored by homonyms. This process involved searching the for the author’s webpage on the web–and then comparing his/her CV with the publication list obtained from the WoS–as well as by comparing the discipline of the researcher with the discipline of the journal in which the paper is published and the institution appearing on the paper with the affiliation of the researcher. Given that the Web of Science database only began indexing the full first names of authors in 2008, this step proved to be quite demanding, and resulted in a reduction of the number of author-paper combinations from more than 375,000 to 15,028 papers, of which 11,980 were citable items (articles, notes and reviews).

In order to take into account the different citation practices of the various specialties in which TED presenters are active [36], the number of citations of each paper was divided by the average number of citations received by all papers of speciality assigned to the journal in which it was published for the same publication year. When this number was greater than one, it indicated that the researcher had, on average, a mean impact above the world average of the specialities where he/she published. Numbers below one implied the converse.

## Results

The results are split into three main sections, corresponding to the three research questions. The first section provides an overview of the demographic characteristics of the presenters, the second reviews the relationship between these characteristics and video popularity, and the third assesses the citation impact of presenting at TED.

### Presenter Demographics

This section reports on results relevant to the first research question, providing a descriptive account of TED presenters, with a focus on gender, age, institution, and online visibility. Information on academic status in terms of citations is presented in later sections.

#### Gender

From the 998 unique individual presenters of the 1,202 TED talk videos examined in this study, 21% were academics (n = 206) and 27% were female (n = 268). There were no statistically significant differences in the distribution of gender by academic status (academic: 158 male, 48 female; non-academic: 572 male, 220 female).

#### Academic age

**Figure 1:**
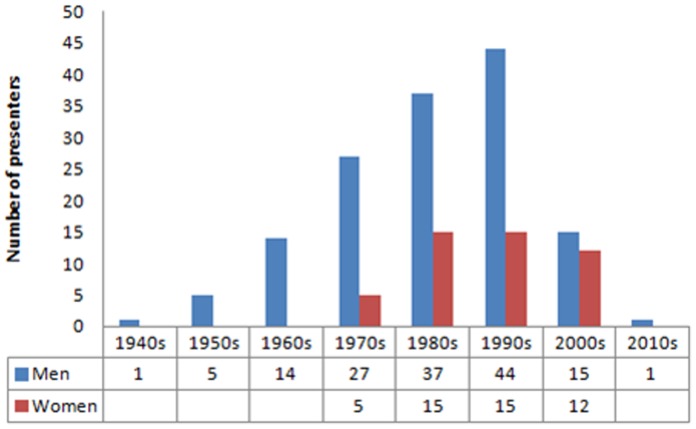
Dates of doctoral degree for academic presenters, by gender.

The date of doctoral degree was located for 191 of the 206 academics. The plurality of presenters received their degrees in the 1990s, and female presenters tended to be younger than their male counterparts. No female presenter received her degree before the 1970s (Figure 1).

Academic rank was identified for 183 of the academics. Of these, the majority (73%; n = 134) were at the professor rank (87 professors, 40 distinguished/named professors, and 7 emeriti). Eighteen were at the associate professor rank, and five were assistant professors. The remaining 26 academics possessed a variety of titles (e.g., adjunct, lecturer, and research scientist).

#### Academic affiliation

Given that a prerequisite for being classified as an academic was an affiliation with an academic institution, university affiliation information was available for all “academics.” These individuals were employed at 99 unique institutions, with 70% of the institutions represented by a sole presenter (Table 1).

**Table 1:** The ten institutions hosting the most TED presenters.

Institution	Presenters
**MIT**	16
**Stanford University**	14
**Harvard University**	13
**Columbia University**	7
**University of Oxford**	7
**University of California, Berkeley**	6
**University of California, Los Angeles**	5
**University of Southern California**	5
**University of Washington**	5
**University College London**	5

More than one-quarter of the presenters (n = 55) were from a California-based institution (representing nearly one-fifth of all institutions in the sample [n = 20]), perhaps reflecting TED’s origins in California; it is also possible that this is due in part to the fact that one of TED’s two main conferences is held in California. In addition to those universities listed in Table 1, Claremont had four presenters, Caltech had three presenters, and UC- Riverside, -San Francisco, -San Diego, and –Santa Barbara all had two presenters. UC- Irvine, -Davis, -Santa Cruz, California College of the Arts, and San Diego State University all had one. The majority of institutions (63%; n = 62) were located in the United States, and the majority of academic presenters were associated with United States-based institutions (75%; n = 160).

#### Online visibility

Online visibility was investigated by conducting an Internet search (using Google) for each academic. Nearly all academics had a website, and 71% had a Wikipedia page about them. An online curriculum vita (CV) could be located for 67% of the academics, as seen in Table 2.

**Table 2:** Online visibility for academic presenters.

	Men (n = 158)	Women (n = 48)	Total (n = 206)
**Website**	148 (94%)	46 (96%)	194 (94%)
**Wikipedia page**	118 (75%)	29 (60%)	147 (71%)
**CV**	101 (64%)	36 (75%)	137 (67%)
**Facebook**	64 (41%)	20 (42%)	84 (41%)
**Twitter**	64 (41%)	21 (44%)	85 (41%)

Facebook and Twitter were the most commonly used social media. Blogs were located for ten of the scholars, and several individuals had Google+ and/or YouTube accounts (not shown). In general, women were more likely to have an online presence than men were (the exception was that males were more likely to have a Wikipedia page dedicated to them). In order to assess whether this was due to the fact that the sampled women tended to be slightly younger (in terms of academic age) than the sampled men, we analyzed public visibility in relation to academic age (Table 3).

**Table 3:** Online visibility for academic presenters by year of doctoral degree.

	<1970 (n = 20)	1970s (n = 32)	1980s (n = 52)	1990s (n = 59)	>1999 (n = 28)
**Website**	18 (90%)	29 (91%)	48 (92%)	56 (95%)	28 (100%)
**Wikipedia page**	20 (100%)	27 (84%)	37 (71%)	41 (69%)	14 (50%)
**CV**	11 (55%)	24 (75%)	34 (65%)	42 (71%)	24 (86%)
**Facebook**	10 (50%)	10 (31%)	20 (38%)	26 (44%)	11 (39%)
**Twitter**	7 (35%)	10 (31%)	19 (37%)	27 (46%)	16 (57%)

It was found that Wikipedia pages are often associated with older academics, while younger academics’ web presences tend to take the form of personal webpages, online CVs, and Twitter usage. There was no clear pattern by age in the use of Facebook. These differences were statistically significant for Wikipedia but not for the other metrics (using an independent samples t-test for Ph.D. date with a Bonferroni correction).

### Video Popularity

The results in this section mainly address the second question, namely, whether there is a relationship between the popularity of a TED video and the gender, academic status, age, or institutional affiliation of its presenter. The figures provided related to all presenters unless it is clear from the context that the results refer only to academic presenters.

#### Gender

To assess whether gender was a significant factor in video impact, the five video popularity indicators (YouTube comments, YouTube views, YouTube like proportion, TED views, TED comments) were compared between males and females using a Mann-Whitney U test (Table 4). After a Bonferroni correction, YouTube views and YouTube like proportions (the ratio of likes to likes plus dislikes for each video) revealed significant differences at the p = 0.01 level (a Bonferroni correction for multiple tests gives p = 0.010 for the p = 0.05 level and p = 0.002 for the p = 0.01 level).

**Table 4:** Mann-Whitney U-test for gender (679 males, 257 females).

	TED site views	TED site comments	YouTube Views	YouTube Comments	YouTube Like Proportion
**Male median**	416632	121	52981	191	0.9469
**Female median**	378747	129	39320	228	0.9092
**Significance, p = **	0.079	0.081	0.000	0.115	0.000

Videos by male presenters were more frequently watched than those by female presenters on YouTube, but the same was not true for the TED website. There was no significant gender difference in terms of the number of comments received. Nevertheless, male-authored videos featured a significantly higher like proportion on YouTube.

#### Academic status

To judge whether the status of a presenter as an academic was significant, the five video popularity indicators were analyzed using a Mann-Whitney U test (Table 5). A Bonferroni correction for multiple tests gives p = 0.010 for the p = 0.05 level and p = 0.002 for the p = 0.01 level.

**Table 5:** Mann-Whitney U-test for being an academic (736 not academic, 202 academic).

	TED Site Views	TED Site Comments	YouTube Views	YouTube Comments	YouTube LikeProportion
**Non-acad. median**	390473	120	46469.5	188.5	0.9330
**Acad. median**	439112	150.5	53469	267.5	0.9505
**Significance, p = **	0.172	0.003	0.012	0.000	0.000

Academic presenters attract more comments in the TED and YouTube websites and have a higher like proportion on YouTube. There is no evidence that they are watched more on either site.

#### Academic age

To gauge the impact of academic age, the year in which individuals received their Ph.D. was correlated against the five video popularity indicators. None of the correlations are significant at p = 0.05 (Table 6), suggesting that academic age has little impact on popularity of any kind.

**Table 6:** Spearman correlations between Ph.D. award year and various popularity statistics (n = 263).

Metric	TED Site Views	TED Site Comments	YouTube Views	YouTube Comments	YouTube LikeProportion
**Ph.D. date**	−0.076	0.013	−0.074	−0.027	0.004

#### University affiliation status

The rank of an affiliated university was correlated against the five video popularity indicators. Universities outside of the top 200 (according to the Times Higher Education World University Rankings 2011–12) were allocated a joint last rank (201). No significant differences (at the p = 0.05 level) were found (Table 7).

**Table 7:** Spearman correlations between affiliated university rank and various popularity statistics (n = 197).

Metric	TED Site Views	TED Site Comments	YouTube Views	YouTube Comments	YouTube Like Proportion
**University status**	−0.033	0.004	−0.033	0.034	−0.056

#### Citation analysis

As noted in the introduction, elite scholars have typically been associated with science popularization activities. To decide whether high-impact (in terms of citations and publications) scientists were associated with more popular videos, we correlated the relative citation impact (excluding self-citations) of a scholar’s publications and the total number of Web of Science (WoS) documents produced by them with YouTube and TED popularity statistics (Table 8).

**Table 8:** Spearman correlations between total WoS documents or relative impact scores (excluding self-citations) and popularity metrics.

	YouTube Views	YouTube Comments	YouTube Like proportion	TED Comments	TED Views
**WoS documents**	0.097	0.067	0.241**	−0.036	0.059
**Relative Impact Score**	0.171	0.128	0.084	0.066	0.132

** Significant at p = 0.01, Bonferroni corrected for n = 12 from 0.01 to 0.00083. Other figures are not significant at p = 0.05. – N = 197 for YouTube and n = 206 for the TED site.

77% of the academic TED presenters had a relative impact score above average (1) for the journals in which they published, and the mean impact of TED presenters’ papers taken together was more than three times the world average. Similarly, 74% were affiliated with a top 200 university, suggesting that they are high-impact scientists. Nevertheless, Table 8 shows that their relative impact did not correlate with the popularity of their TED talk. The only publication statistic that correlated with anything was the total number of documents in WoS. This correlation with YouTube like proportions may indicate that talks given by natural or medical scientists were more popular than those by others, as researchers in those disciplines typically contribute to more papers when one uses full counting [37], as is the case in this paper.

#### The impact of TED Talks on academic presenters

Gingras and Wallace found that academics who received a Nobel Prize saw a subsequent increase in citations to their articles [38]. Following this example, in order to evaluate whether presenting at TED leads to increased citations for academics, we compared the number of citations to an academic’s publications before and after the talk for the three years for which sufficient data were available. Such evidence might suggest that science popularization could serve as an incentive mechanism, in that it generates academic capital for the scientists. Table 9 displays the median citations (excluding self-citations) received by academics in the years immediately before and after their first TED video was published (TED-2 indicates two years before the TED presentation) as a percentage of all citations received by the academic. Scientists with fewer than 10 total citations are excluded. All scientists included had received at least one citation on or before TED - 2.

**Table 9:** Median citations received by academics in the years immediately before and after their first TED talk.

TED year	Scientists	TED -2	TED -1	TED	TED +1	TED +2	TED +3	TED +4
**2009**	32	7.6%	7.0%	8.5%	8.5%	7.4%		
**2008**	28	5.5%	6.6%	6.8%	6.8%	7.2%	6.7%	
**2007**	18	5.3%	7.0%	7.7%	10.5%	9.9%	12.3%	14.2%

The table suggests that TED presentations do not trigger significant increases in citations for an academic. Assuming that academics don’t receive citations in the year that they gave their TED presentation due to normal publication delays, only for 2007 did citations increase significantly after the TED presentation (nearly doubling four years later). However, this is based upon only 18 academics, and these academics’ citations were already increasing before their TED presentation.

## Discussion and Conclusions

In regard to the first research question, the majority of presenters of the investigated 1,202 TED videos were male (73%) non-academics (79%). This suggests that science popularization is only a small part of the function of TED talks, which includes presentations by technologists, designers, and entertainers. However, introducing scholars on the same stage possibly mediates the way in which these academics present their work. Of the academic presenters, females tended to be younger (in terms of academic age) than males. Academic presenters were typically senior faculty (73% at professor rank), from United States-based institutions (75%), featured on a Wikipedia page (71%), and cited more frequently than average (77%). The fact that academics featured on TED also tended to be the successful elite fits the traditional demographic previously found for successful science communicators [14], [15], [18], [19]. However, it is notable how few academics comprise the pool of TED Talk presenters.

In regard to the second research question, male-authored TED Talks on YouTube (but not on the TED website) were more popular and more liked than TED Talks by women. This could be in part due to the nature of YouTube’s audience; although the gender balance is the same as for the Web as a whole [39], [40]; female viewers may be less influential, given that they seem less likely to comment than males are [1].

Videos by academics were more commented on than videos by others, although there was little difference in how often they were viewed. Within the academic group, there were no significant differences in popularity metrics by academic age or university affiliation status. We propose three alternative explanations for this: 1) University affiliations do not register with the online audience, 2) University prestige is irrelevant to the online audience, or 3) University prestige is relevant, but this factor is offset by academics at less prestigious universities having to perform better to be invited to present at TED or to have their video be published online (a curatorial decision by TED is made regarding which conference talks are “published” as TED Talks [25]).

In regard to the third research question, giving a TED presentation appeared to have no impact on the number of citations subsequently received by academic presenters. This suggests that either TED does not promote the work of scientists within their own community or that the positive impact of publicity is offset by any negativity that accrues due to the tendency of fellow researchers to question the presenter’s motivations.

The above findings can help to shed light on some theories and beliefs about science popularization. Research on science communication argues that “media foster negative perceptions of science and technology and that the public, because of a widespread lack of science literacy, is relatively defenceless to the media’s influence” [11]. The findings from this study run counter to this argument. Despite being in a minority, videos with academic presenters were preferred. This demonstrates positive associations with science and technology information and also a possible level of discernment between presentations made by academics and those by other public figures. However, an alternate explanation could be that academic presenters are less controversial than non-academics and therefore less likely to accrue negative popularity metrics.

The blurring of lines between academics and journalists has long been a point of discussion in science communication; take, for example, Bourdieu’s notion of the journalist-academic [41]. However, given the “battery of communicative options” in the scholarly communication ecosystem, there is renewed concern that assiduous self-promotion by certain scientists will lead to unwarranted prestige for those engaging in these venues [42]. The academic presenters in this study were fairly visible online and academically elite, as demonstrated through their citation counts. It has been suggested that “Web video opens a new form of public intellectualism to scholars looking to participate in an increasingly visual culture” [43]. That the Matthew effect [44] would function in the online environment is highly likely; scholars have noted that online indicators serve as attention metrics that feed into the behaviors of both audience and presenter [45]. An economy of attention prevails in academia, and “positive and immediate online affirmation may incentivize scholars to engage in this environment” [16]. However, although it may lead to greater visibility online, there is no evidence that participating in TED Talks lead to an increase in the traditional metric of academic capital: citations. In contrast, it is quite possible that TED’s academic presenters are often chosen partly *because* they are recognized scholars in their fields.

One of the limitations of this research is the partly unknown audience for TED videos. As suggested by Millstone and van Zwanenberg, “there is not simply one ‘public’…our societies are culturally, regionally, socially and economically diverse” [34]. It is altogether possible that those who watched and “liked” these videos were often themselves academics. Figures from Alexa.com suggest that the TED audience is young and well-educated, with the age range 18–24 and the education status “Graduate School” being overrepresented amongst ted.com visitors compared to the rest of the Web [46]. Future research should seek to discern more details about the audience for a more comprehensive interpretation of impact measures. This could be done unobtrusively via an analysis of the comments and other traces left when interacting with the platforms. On a more obtrusive level, one could sample and survey viewers.

Future research should also seek to understand how TED presentations (and online videos in general) contribute to the public’s perception of science. In the late 1990s, 75% of scientists agreed with the sentiment that “the media, when covering science, are more interested in sensationalism than truth [and] that media coverage concentrates too much on trend discoveries rather than basic research and development” [47]. In 2002, Nisbet et al. proposed a media effects model that demonstrated associations between the type of media used to consume scientific knowledge and perceptions of science [11]. Given the dramatic changes in mediatization in the last decade [48], it may be time to reassess the ways in which the public consumes scientific information and the relationship between these modes of consumption and subsequent perceptions and knowledge of science.
